# Neutropenia induced by several second-generation antipsychotics :A case report

**DOI:** 10.1192/j.eurpsy.2023.2138

**Published:** 2023-07-19

**Authors:** H. Zarouf, M. Chtibi, S. Belbachir, A. Ouanass

**Affiliations:** 1 Ar-razi University Psychiatric Hospital, Salé, Morocco

## Abstract

**Introduction:**

Antipsychotic medications remain the mainstay of the treatment of various psychiatric disorders, particularly schizophrenia. However, this therapeutic class can induce a range of side effects. Although the treatment with second generation antipsychotics includes a lower risk for extrapyramidal symptoms as compared to first generation antipsychotics, there are numerous adverse events that can result from atypical antipsychotics. Since the introduction of clozapine, there has been increased awareness regarding antipsychotic-induced hematological side effects.

**Objectives:**

The objective of this case report is to highlight the importance of the management of antipsychotic-induced neutropenia.

**Methods:**

We report a patient with history of schizophrenia who developed neutropenia induced by Haloperidol, Chlorpromazine, Olanzapine, Amisulpride and Aripiprazole.

**Results:**

We present a case of a 43-year-old male patient with a history of schizophrenia, admitted in our department for the management of a state of agitation in the context of a relapse of his condition. On admission, the patient experienced psychotic symptoms, including delusions and auditory hallucinations, in addition to negative symptoms, such as affective flattening, alogia, avolition and asociality. He was then started on 12 mg of Haloperidol and 200 mg of Chlorpromazine with a white blood cells count (WBC) of 5.98 x 10^9^/L and absolute neutrophil count (ANC) of 2.52 x 10^9^/L (WBC reference range: 4.0-10.0 x 10^9^ /L; ANC reference range: 1.5-7.0 x 10^9^ /L). The patient did not report adverse events on this medication.

15 days into hospitalization, a mild neutropenia was detected (WBC=3.92 x 10^9^ /L and ANC=1.01 x 10^9^ /L), leading to a discontinuation of the antipsychotic treatment. No signs of infection were found. After one month, the patient had a normal WBC and ANC. Aripiprazole was discussed as a first alternative and was begun at 5 mg/day and then at 10 mg/day. After one week of treatment with Aripiprazole, the patient’s WBC was normal, but the ANC decreased again leading to a moderate neutropenia (ANC=0.91x 10^9^ /L). The antipsychotic treatment was once again discontinued and the hematological evaluation found no other identifiable cause. Afterwards, neither Olanzapine nor Amisulpride showed significant response to this adverse effect. Finally, the administration of Risperidone led to a positive outcome on the WBC and the ANC.

**Conclusions:**

Awareness regarding the hematological side effects of antipsychotics should increase and clinical management of this type of adverse event should be a subject of interest among psychiatrists.

**Disclosure of Interest:**

None Declared

